# Supplementary material for: Targeted calcium influx boosts cytotoxic T lymphocyte function in the tumour microenvironment
Source: Nat Commun. 2017 May 15;8:15365. doi: 10.1038/ncomms15365 (PMC5440668; doi:10.1038/ncomms15365)
Supplement: Supplementary Information — Supplementary Figures [file ncomms15365-s1.pdf]

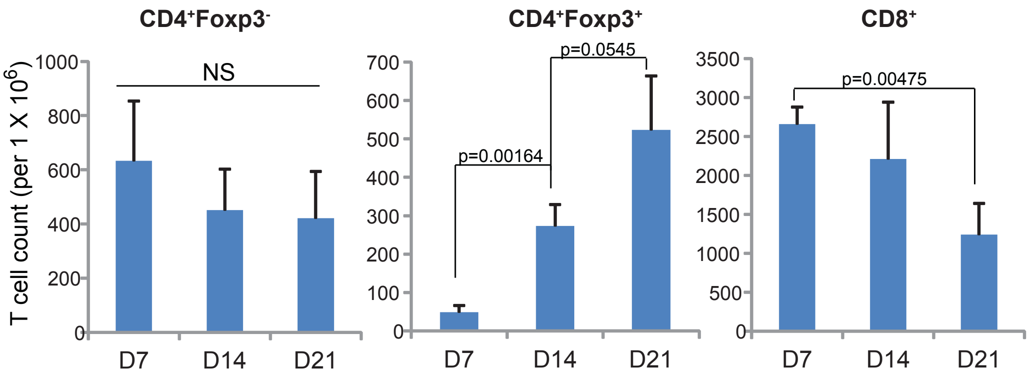

Supplementary Figure 1. T cell infiltration in the B16F10 melanoma. B16F10 melanoma cells were intradermally injected into the mouse ear skin. Tumor samples were collected on days 7, 14 and 21 (n > 5). $CD4^+$ $Foxp3^-$ T cells, $CD8^+$ T cells and $CD4^+Foxp3^+$ Treg cells were monitored by flow cytometry. Data were analyzed with Student's *t*-test.

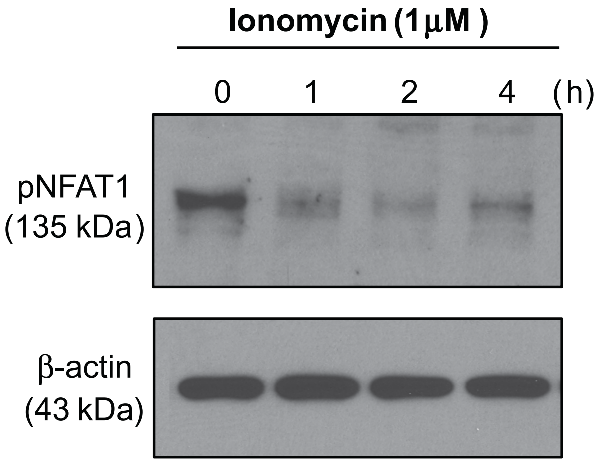

Supplementary Figure 2. Treatment of OT-I CTLs with ionomycin resulted in the dephosphorylation of NFAT1. OT-I CTLs were stimulated by 1 μM ionomycin for indicated times, and lysed in RIPA buffer. The cell lysates were analyzed by immunoblotting using anti-phospho-NFAT1 or anti-β-actin.

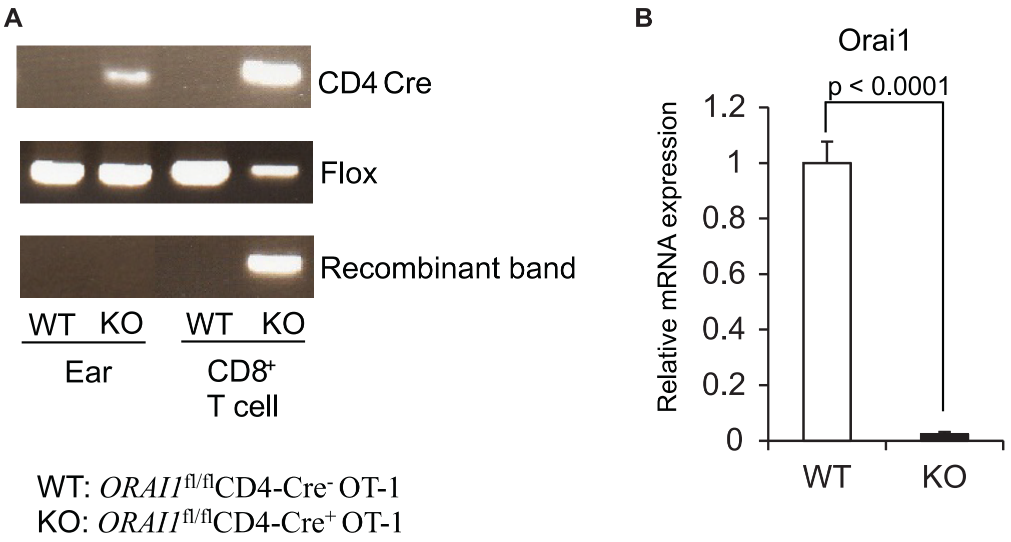

Supplementary Figure 3. Genotypic characterization and identification of CD4-cre Orai1 conditional knockout mice. (a) Genomic DNA samples were prepared from the mice ears and purified $CD8^+$ T cells, and PCR was performed using primers for the Cre gene sequence, intact Orai1-floxed allele and the recombined Orai1 allele. (b) The mRNA expression level of Orai1 was analyzed by quantitative RT-PCR (n = 3). Data were analyzed with Student's *t*-test.

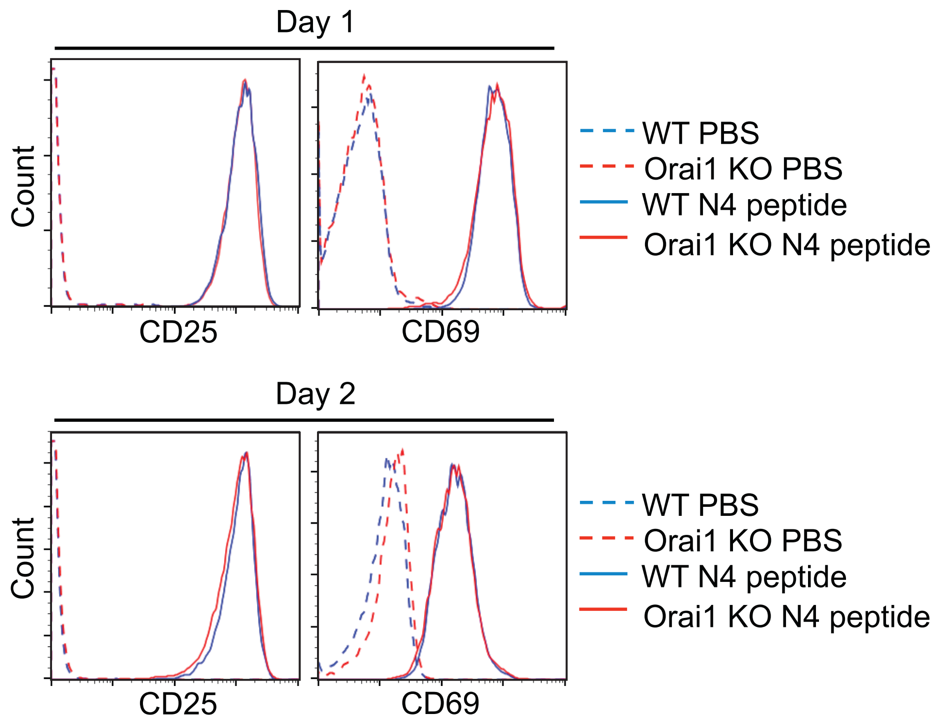

Supplementary Figure 4. Activation of WT and Orai1 cKO OT-I CTLs after N4 peptide stimulation. Purified OT-I $CD8^+$ T cells were stimulated with N4 peptide and irradiated splenocytes. Expression of CD25 and CD69 were measured by flow cytometry.

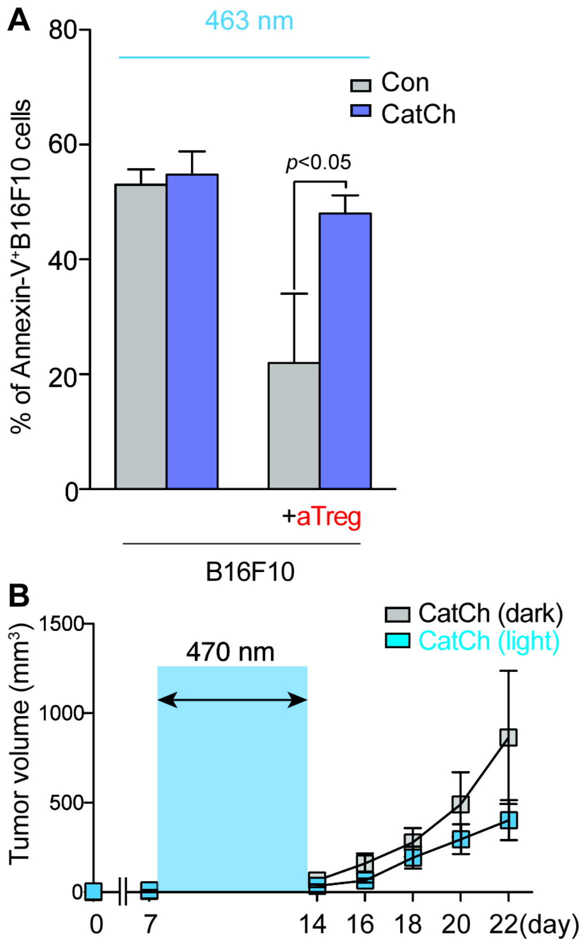

Supplementary Figure 5. (a) Killing of hgp100$_{25\text{–}33}$ peptide-loaded B16F10 cells after light stimulation of CatCh- or GFP-expressing Pmel-1 CD8^{+} T cells for 16 h in the presence or absence of aTregs (n = 4). (b) Growth curves of B16 tumors on the ear treated with adoptive transfer of CatCh-expressing Pmel-1 CD8^{+} T cells with vaccination with mgp100$_{25\text{–}33}$ (EGSRNQDWL) (light, n = 5; dark, n = 6). The light stimulation (470-nm) times are denoted with a blue box. Data were analyzed with Student's *t*-test.

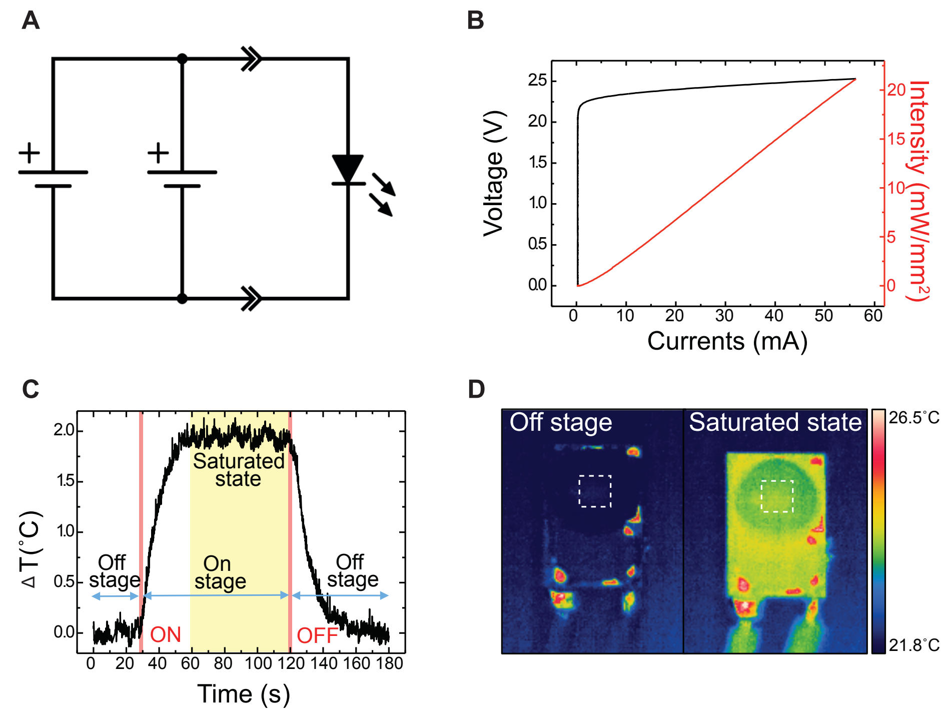

Supplementary Figure 6. Battery powered LED operation and its electrical, optical, and thermal properties. (A) A scheme of battery powered LED system. (B) Light output-current and voltage (LIV) measurement for a 505 nm LED. (C) Temperature variation of the LED system for 90 second operation. (D) Measured temperature distribution for the LED system at off and on (saturated) stage.

Figure 1j: phospho-NFAT1 western blot

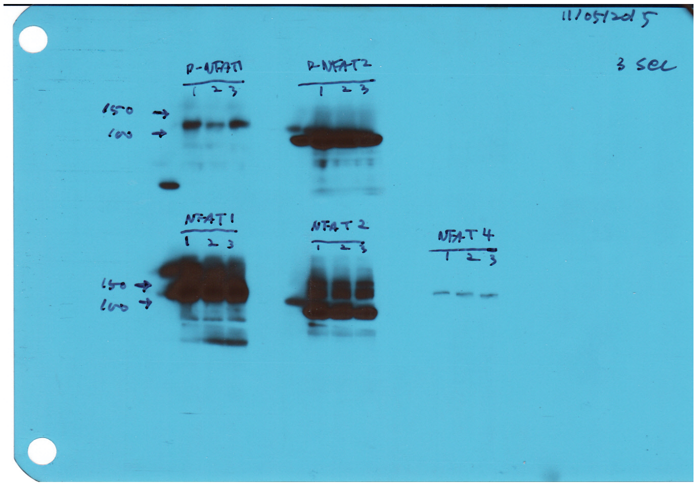

Figure 1j: NFAT1 western blot

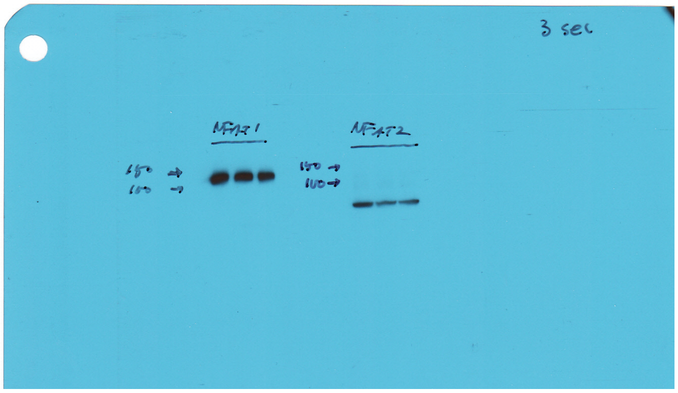

Figure 3b: Phospho-NFAT1 and beta-actin western blot

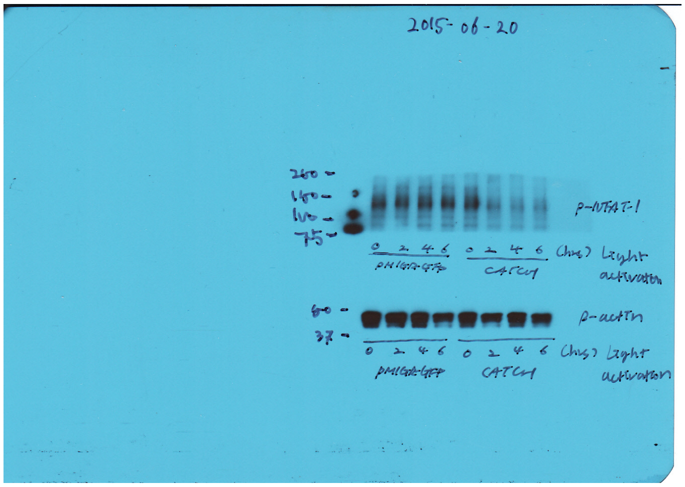

Supplementary Figure 7. Uncropped Western blots for shown in the manuscript.
